# Supplementary material for: Induction of DR5-Dependent Apoptosis by PGA2 through ATF4-CHOP Pathway
Source: Molecules. 2022 Jun 13;27(12):3804. doi: 10.3390/molecules27123804 (PMC9230093; doi:10.3390/molecules27123804)
Supplement: Supplementary file 1 [file molecules-27-03804-s001.zip › Densitometry_Graph.pdf]

Figure 1B

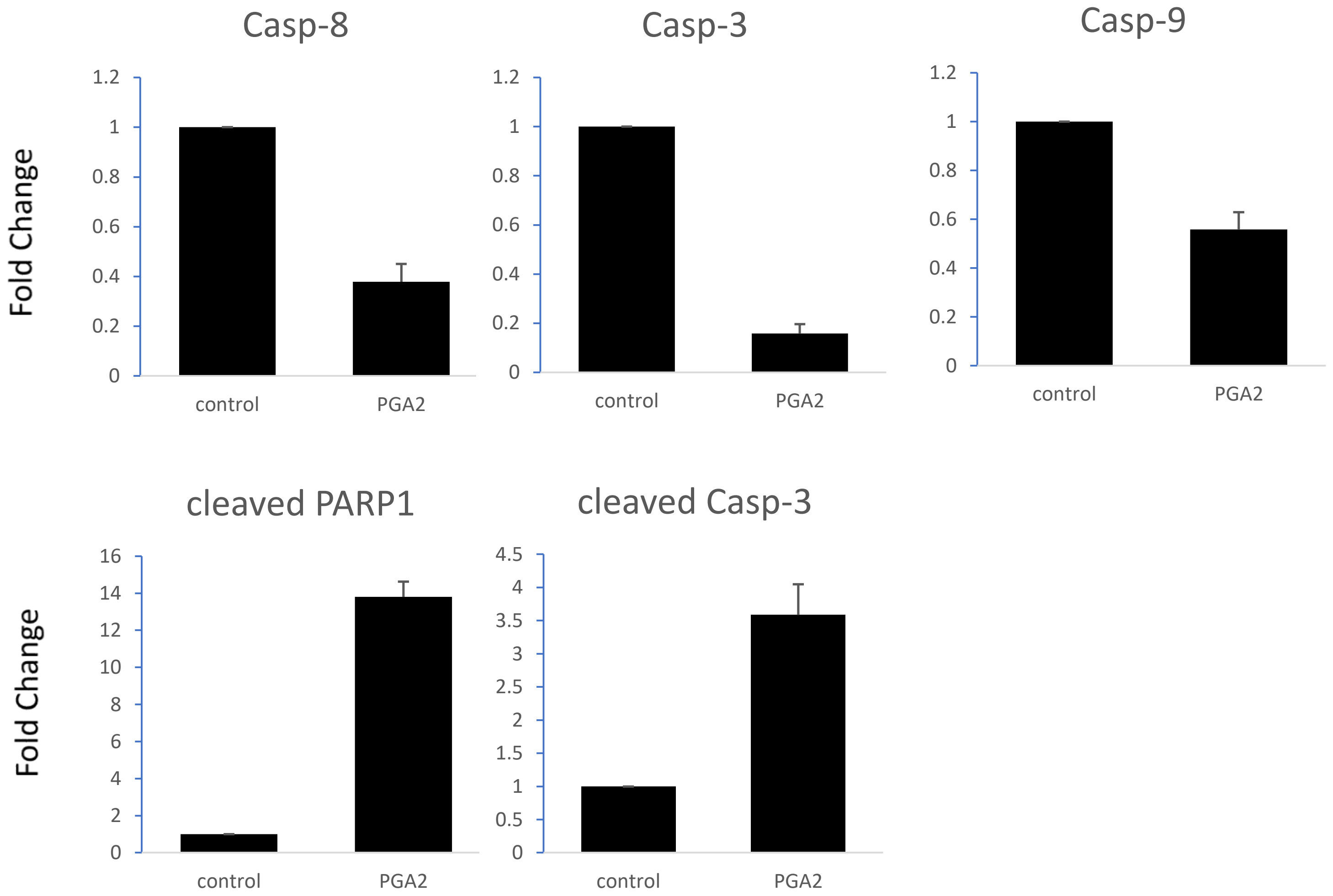

Casp-8
Casp-3
Casp-9
Fold Change
1.2
1
0.8
0.6
0.4
0.2
0
control
PGA2
cleaved PARP1
cleaved Casp-3
Fold Change
16
14
12
10
8
6
4
2
0
4.5
4
3.5
3
2.5
2
1.5
1
0.5
0
control
PGA2

Figure 2A

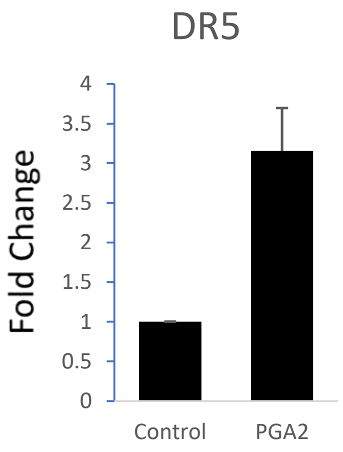

Figure 2C

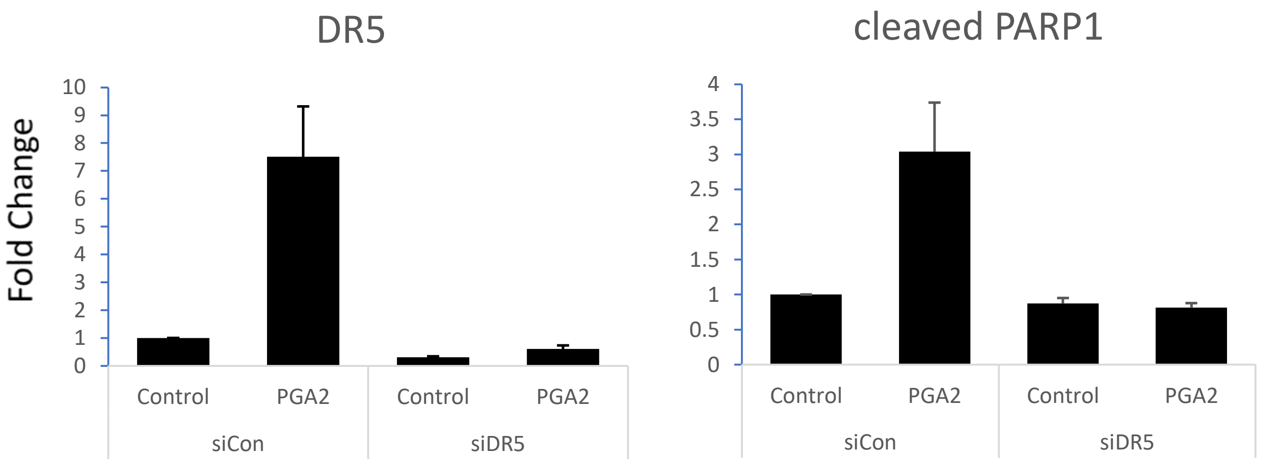

Figure 3A

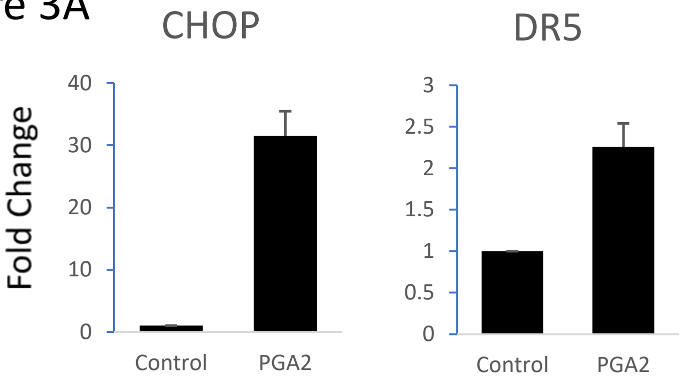

Figure 3B

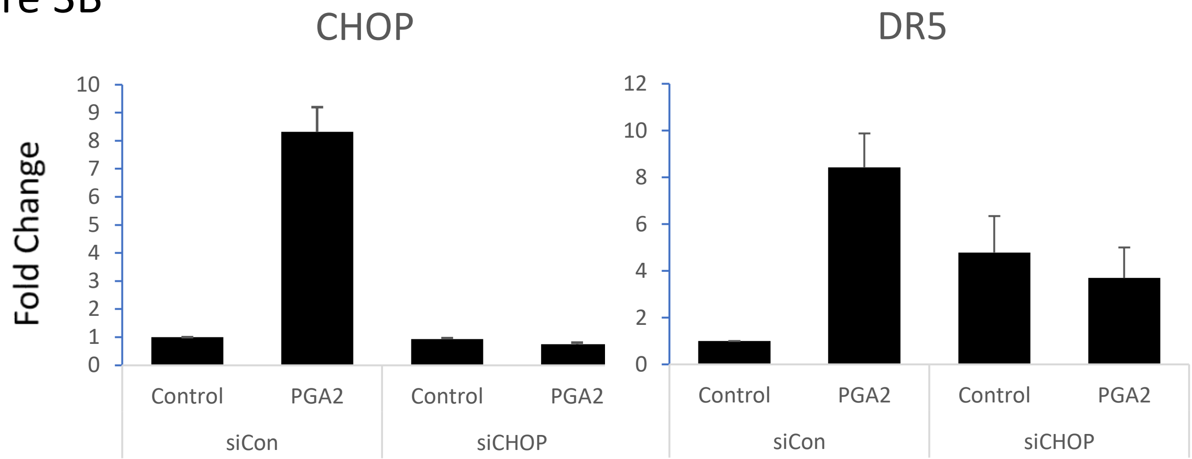

Figure 3C

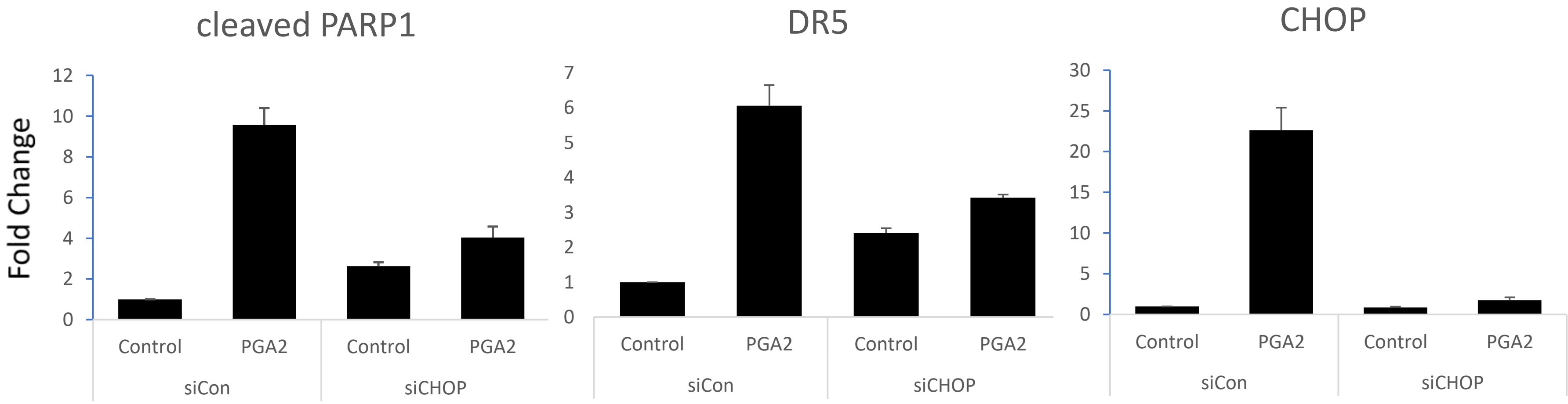

Figure 4B

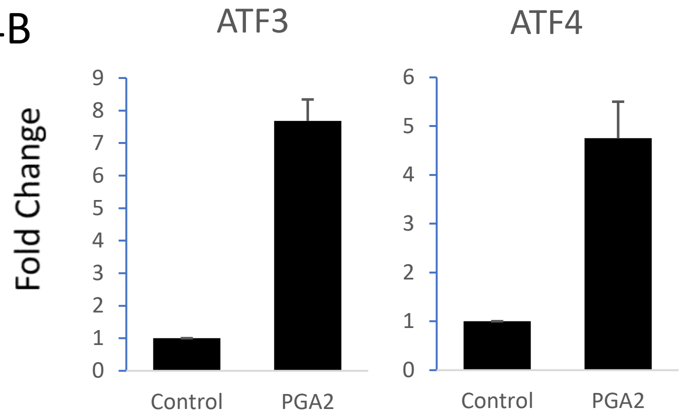

Figure 4D Left

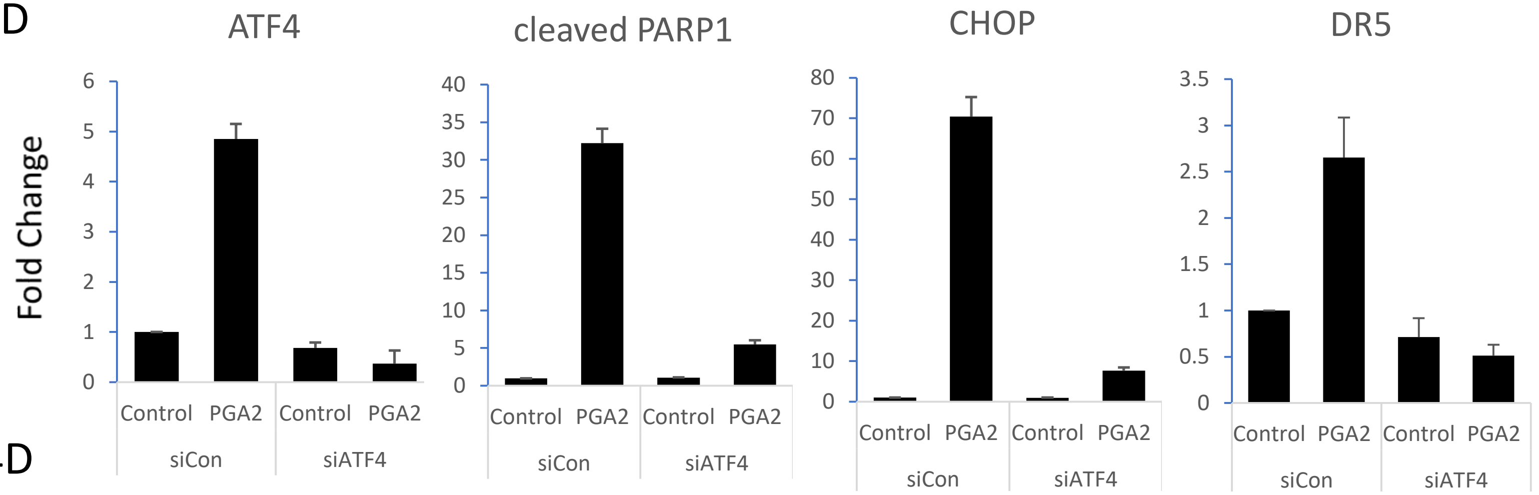

Figure 4D Right

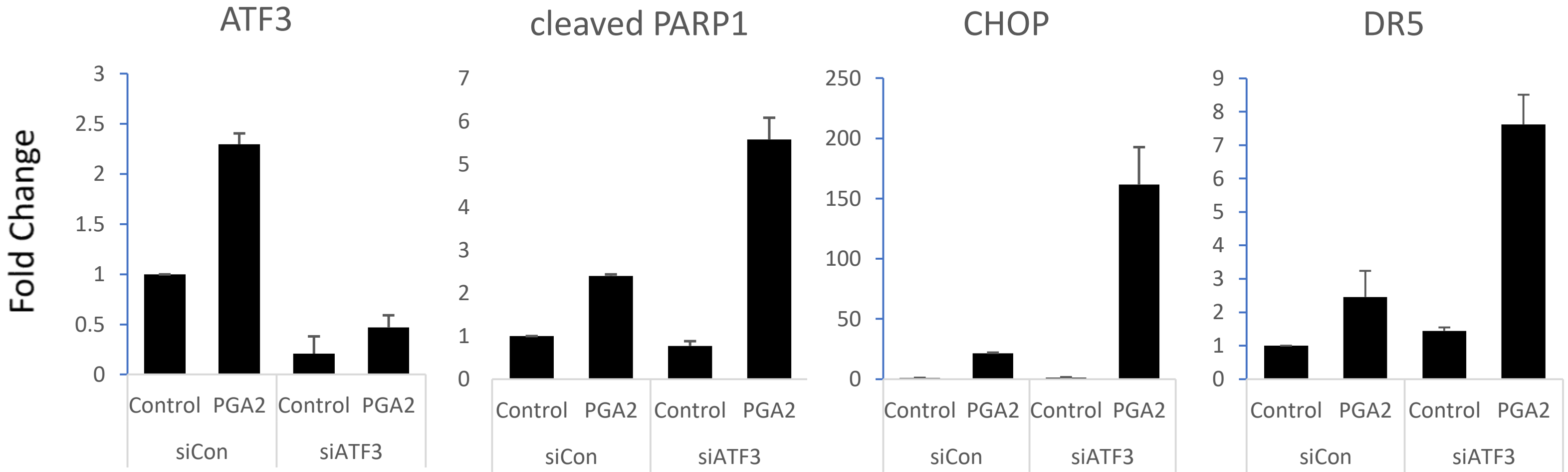

Figure S1

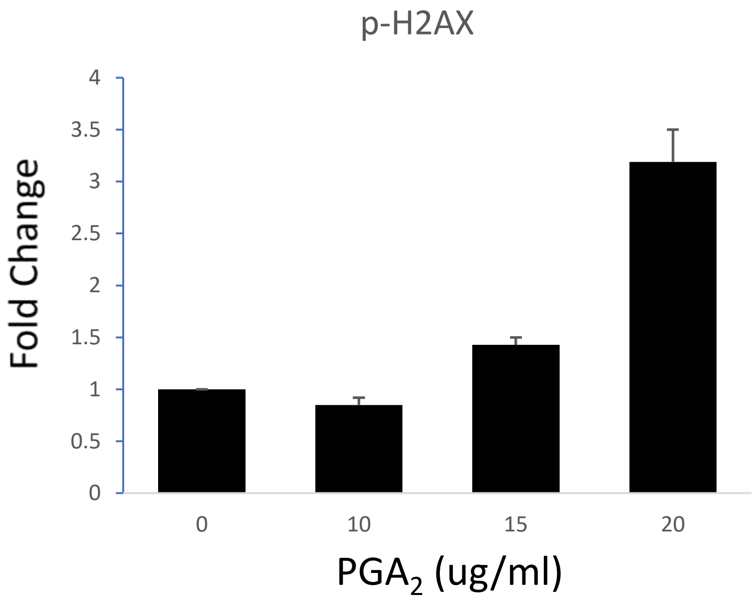

Figure S2

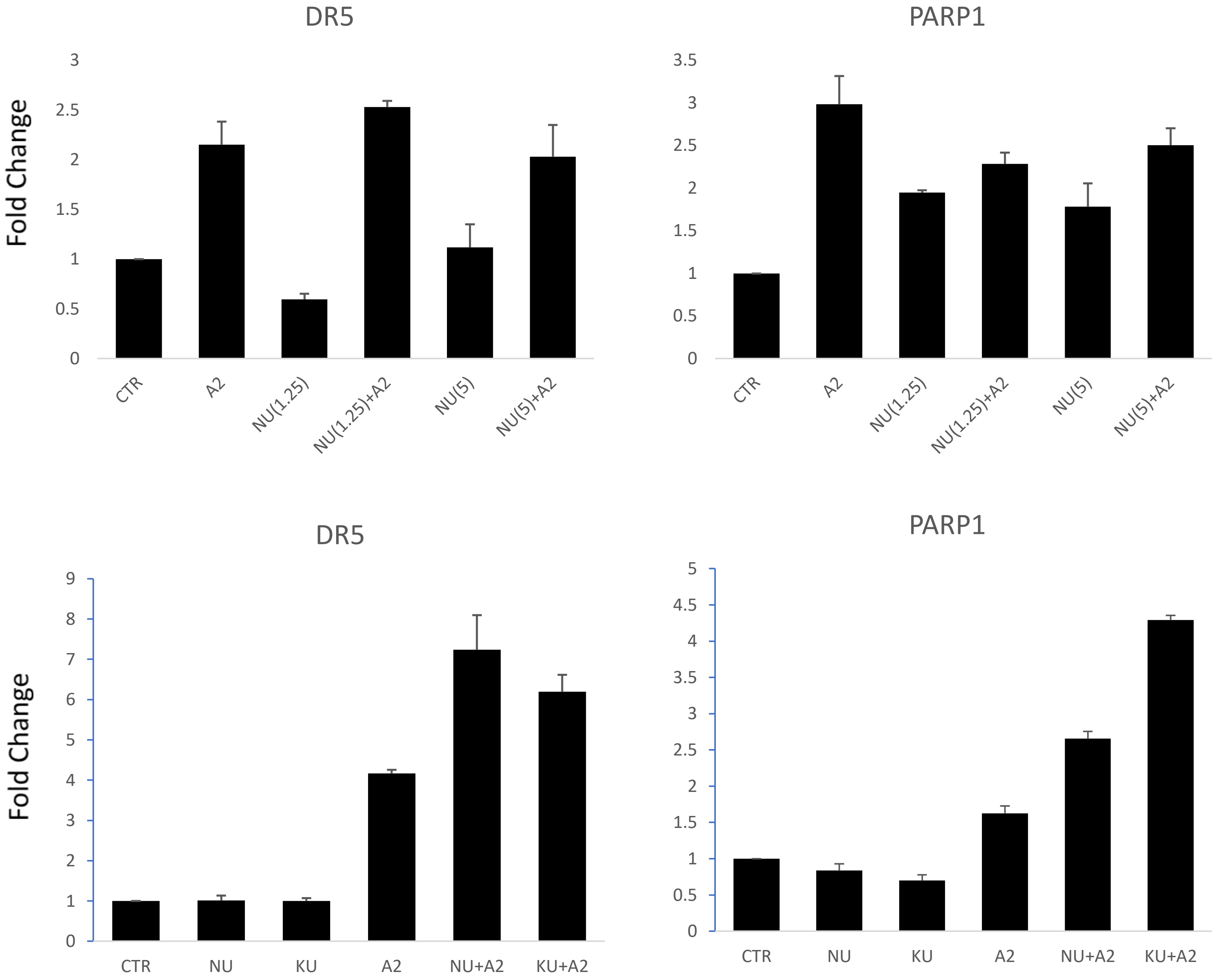
DR5
Fold Change
3
2.5
2
1.5
1
0.5
0
CTR
A2
NU(1.25)
NU(1.25)+A2
NU(5)
NU(5)+A2
PARP1
3.5
3
2.5
2
1.5
1
0.5
0
CTR
A2
NU(1.25)
NU(1.25)+A2
NU(5)
NU(5)+A2
DR5
Fold Change
9
8
7
6
5
4
3
2
1
0
CTR
NU
KU
A2
NU+A2
KU+A2
PARP1
5
4.5
4
3.5
3
2.5
2
1.5
1
0.5
0
CTR
NU
KU
A2
NU+A2
KU+A2

Figure S3

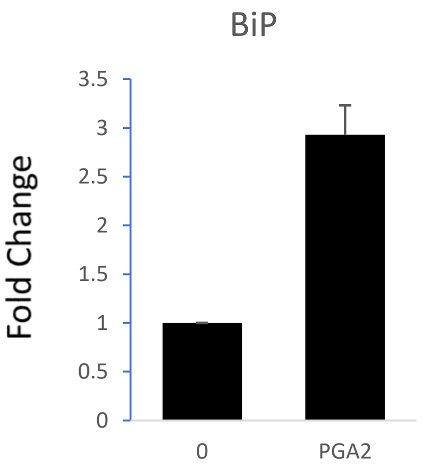

Figure S4

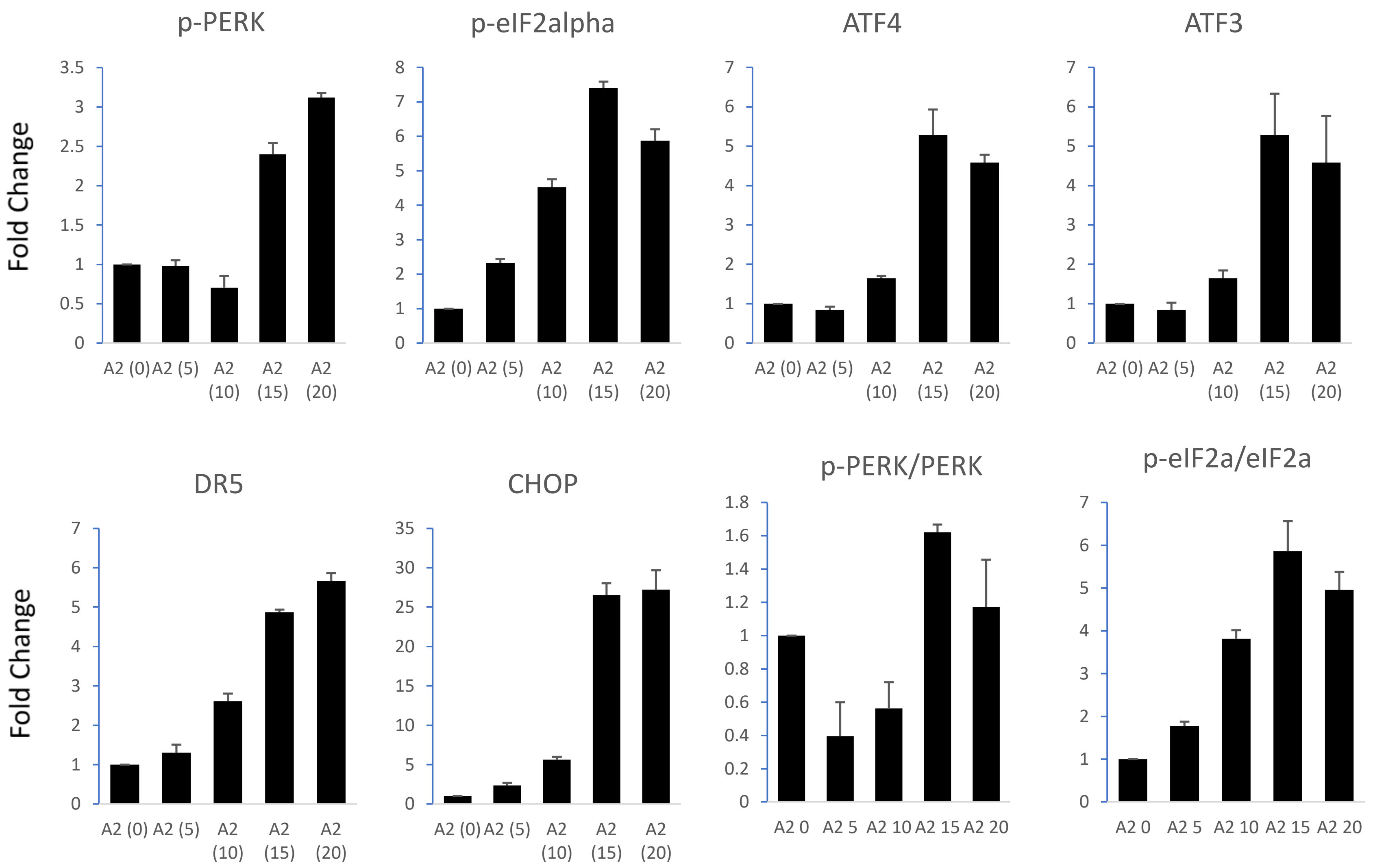

p-PERK
p-eIF2alpha
ATF4
ATF3
Fold Change
3.5
3
2.5
2
1.5
1
0.5
0
8
7
6
5
4
3
2
1
0
A2 (0) A2 (5) A2 (10) A2 (15) A2 (20)
DR5
CHOP
p-PERK/PERK
p-eIF2a/eIF2a
35
30
25
20
15
10
5
1.8
1.6
1.4
1.2
1
0.8
0.6
0.4
0.2
0
A2 0 A2 5 A2 10 A2 15 A2 20

Figure S5

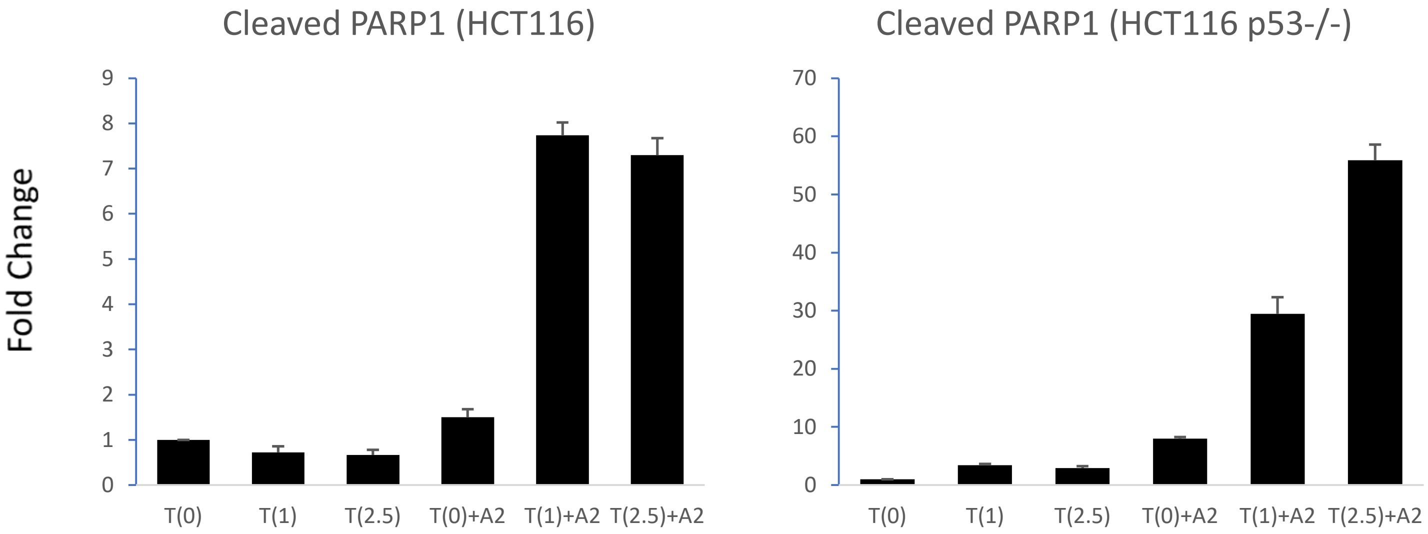
Cleaved PARP1 (HCT116)
Cleaved PARP1 (HCT116 p53-/-)
Fold Change
0
1
2
3
4
5
6
7
8
9
0
10
20
30
40
50
60
70
T(0)
T(1)
T(2.5)
T(0)+A2
T(1)+A2
T(2.5)+A2
T(0)
T(1)
T(2.5)
T(0)+A2
T(1)+A2
T(2.5)+A2
