# Supplementary material for: Induction of DR5-Dependent Apoptosis by PGA2 through ATF4-CHOP Pathway
Source: Molecules. 2022 Jun 13;27(12):3804. doi: 10.3390/molecules27123804 (PMC9230093; doi:10.3390/molecules27123804)
Supplement: Supplementary file 1 [file molecules-27-03804-s001.zip › Supplementary Figures and Legends_KM Park et al.pdf]

Supplementary Figure Legends

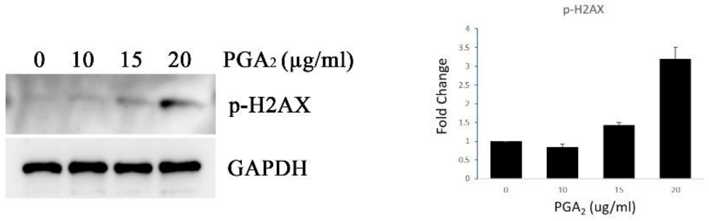

Figure S1. Phosphorylation of H2AX induced by PGA_2 in HCT116 p53 −/− cells. Whole cell lysates of HCT116 p53 −/− cells treated with vehicle or indicated concentrations of PGA_2 for 30 h were subjected to immunoblot analysis against phospho-H2AX (p-H2AX) and GAPDH as the normalizer (left). Densitometric measurement of three independent immunoblot analyses was presented as mean ± SEM (right).

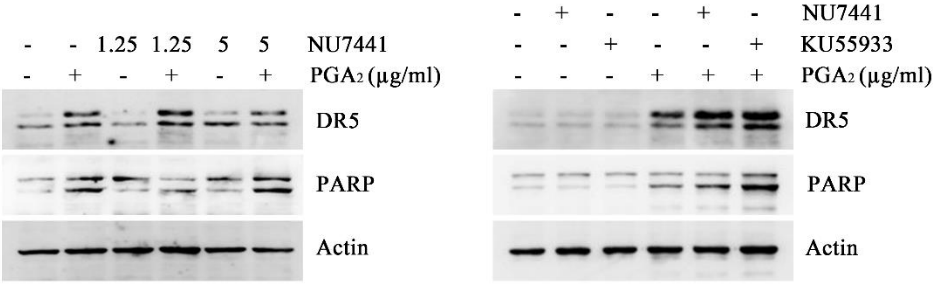

-Densitometry

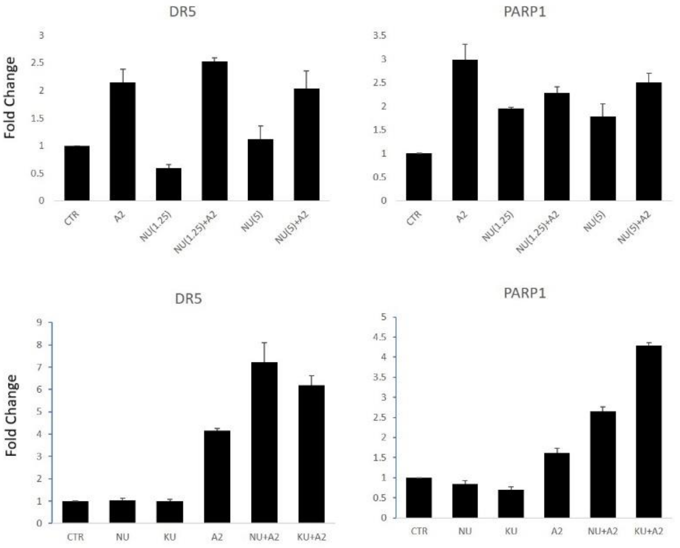

Figure S2. The effect of chemical inhibitors of DNA damage sensing protein kinases on PGA_2-induced *DR5* expression in HCT116 p53 −/− cells. HCT116 p53 −/− cells incubated in the presence of vehicle, NU7441 (5 μM) or KU55933 (5 μM) for 1 h were treated with vehicle or 15 μg/ml of PGA_2 for another 30 h. Cells were then subjected to immunoblot analysis against DR5 and PARP1 using β-actin as an internal reference protein. Densitometric measurement of three independent immunoblot analyses was presented as mean ± SEM.

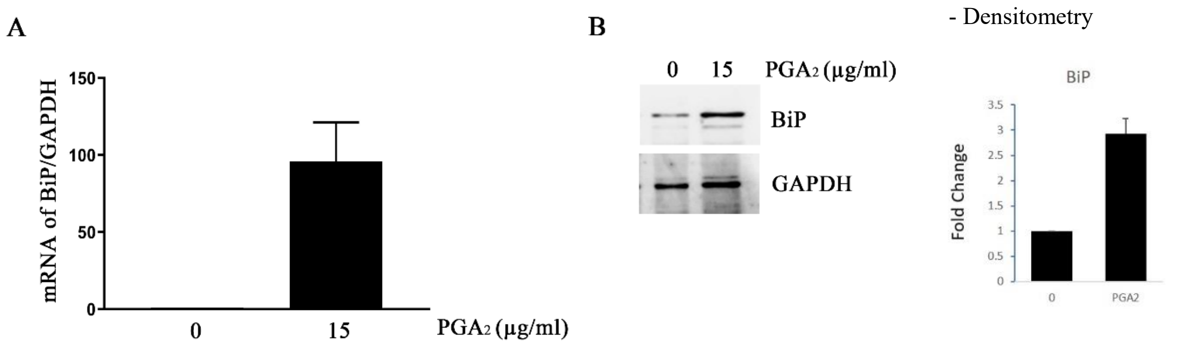

Figure S3. Increase of *BiP* expression by PGA_2 in HCT116 p53 −/− cells. HCT116 p53 −/− cells were treated with vehicle or PGA_2 (15 μg/ml) for 30 h. Total cellular RNAs and whole cell lysates were then subjected to quantitative real time RT-PCR against *BiP* along with *GAPDH* as an internal reference gene (A) and immunoblot analysis against BiP protein and GAPDH protein used as the normalizer (B), respectively. Densitometric measurement of three independent immunoblot analyses was presented as mean ± SEM.

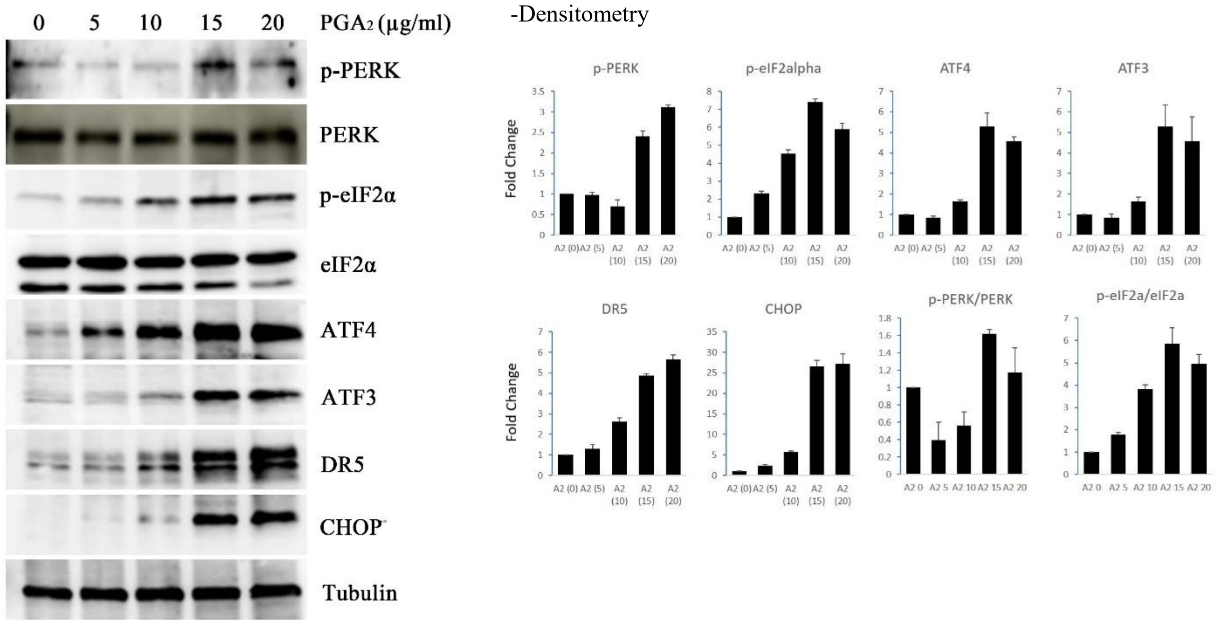

Figure S4. Increase of ER-stress proteins induced by PGA_2 in HCT116 p53 −/− cells. Whole cell lysates of HCT116 p53 −/− cells treated with vehicle or indicated concentrations of PGA_2 for 30 h were subjected to immunoblot analysis against indicated proteins using tubulin as the normalizer. Densitometric measurement of three independent immunoblot analyses was presented as mean ± SEM.

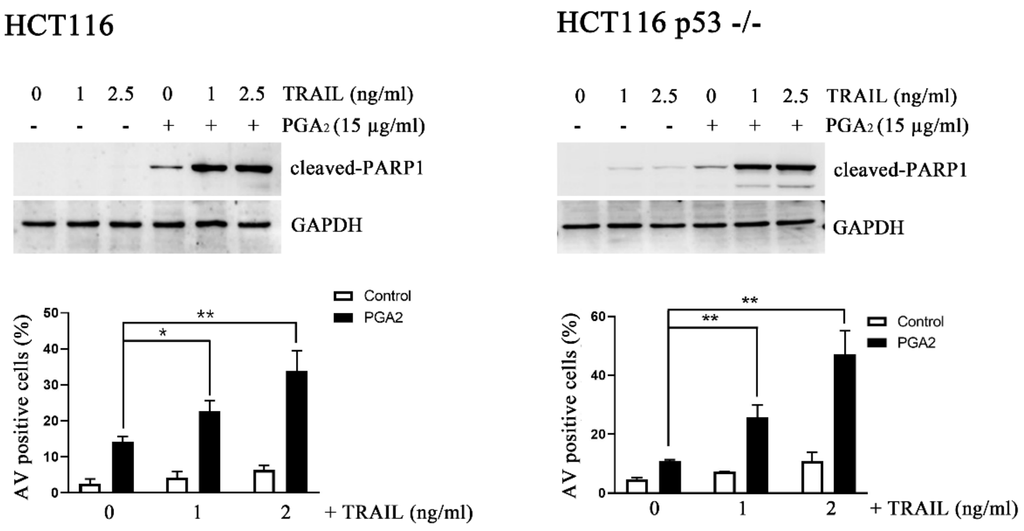

-Densitometry

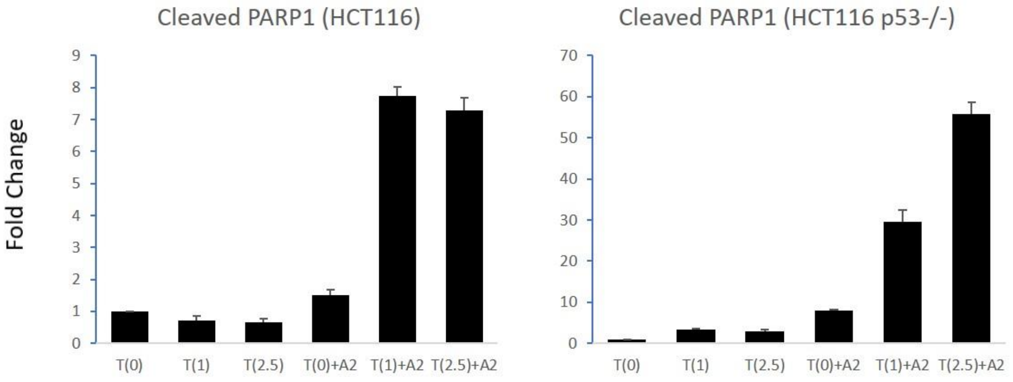

Figure S5. The effect of PGA_2 on TRAIL-induced apoptosis. HCT116 cells (A) and HCT116 p53 −/− cells (B) were treated with vehicle, TRAIL, PGA_2, or a combination of TRAIL and PGA_2. After 15 h, cells were subjected to annexin V (AV) assay and immunoblot analysis against cleaved PARP1 using GAPDH as the normalizer. Densitometric measurement of three independent immunoblot analyses was presented as mean ± SEM.

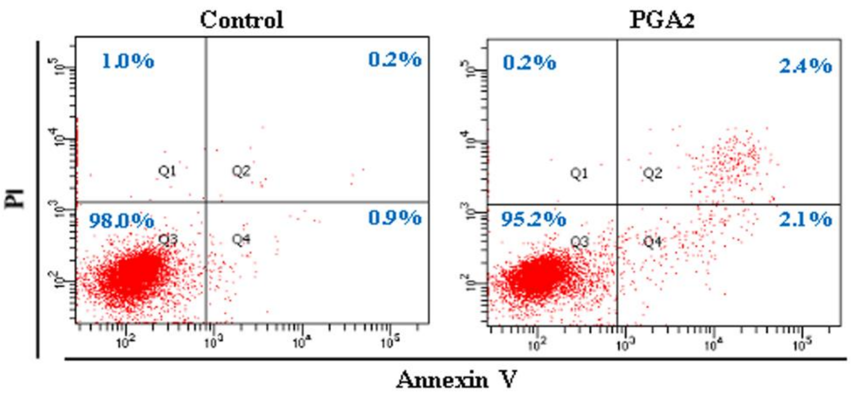

Figure S6. The effect of PGA_2 on the survival of SW620 cells. SW620 cells of which *p53* gene is mutated were treated with vehicle (control) or 15 µg/ml of PGA_2 (PGA_2) for 48 h. Cells were then subjected to annexin V/PI assay.

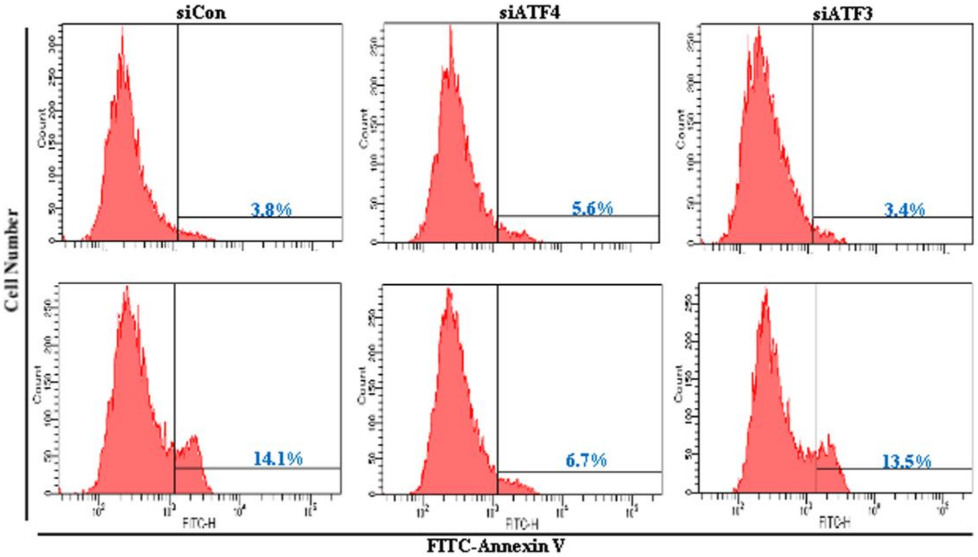

Figure S7. The annexin V (AV) histogram of the results presented in Figure 4. HCT116 p53 −/− cells were transfected with scrambled RNA (siCon) or siRNA targeting ATF3 (siATF3) or ATF4 (siATF4) for 24 h and incubated in the presence of vehicle or PGA_2 (15 µg/ml) for an additional 30 h. Cells were then subjected to annexin V assay. Cell number of 10,000 AV-stained cells was presented as the histogram.
